# Relation between Biofilm and Virulence in *Vibrio tapetis*: A Transcriptomic Study

**DOI:** 10.3390/pathogens7040092

**Published:** 2018-11-26

**Authors:** Sophie Rodrigues, Christine Paillard, Sabine Van Dillen, Ali Tahrioui, Jean-Marc Berjeaud, Alain Dufour, Alexis Bazire

**Affiliations:** 1 Laboratoire de Biotechnologie et Chimie Marines (LBCM), EA 3884, LBCM, IUEM Université de Bretagne-Sud, 56100 Lorient, France; sophie.rodrigues@univ-rouen.fr (S.R.); alain.dufour@univ-ubs.fr (A.D.); 2 UMR6539, Laboratoire des Sciences de l’Environnement Marin (LEMAR), Centre National de la Recherche Scientifique, Institut Universitaire Européen de la Mer, Université de Brest, UBO, IRD, Ifremer, 29280 Plouzané, France; Christine.Paillard@univ-brest.fr; 3 DuPont Nutrition and Health, Danisco France SAS, BP10, F-86220 Dangé-Saint-Romain, France; Sabine.Van.Dillen@dupont.com; 4 Laboratory of Microbiology Signals and Microenvironment LMSM EA 4312, University Rouen-Normandy, 27000 Evreux, France; ali.tahrioui@univ-rouen.fr; 5 UMR 7267, Laboratoire d’Ecologie et Biologie des interactions (EBI), Université de Poitiers, 86000 Poitiers, France; jean-marc.berjeaud@univ-poitiers.fr

**Keywords:** biofilm, virulence, *Vibrio tapetis*, transcriptome, quorum sensing, type VI secretion system, brown ring disease

## Abstract

Marine pathogenic bacteria are able to form biofilms on many surfaces, such as mollusc shells, and they can wait for the appropriate opportunity to induce their virulence. *Vibrio tapetis* can develop such biofilms on the inner surface of shells of the *Ruditapes philippinarum* clam, leading to the formation of a brown conchiolin deposit in the form of a ring, hence the name of the disease: Brown Ring Disease. The virulence of *V. tapetis* is presumed to be related to its capacity to form biofilms, but the link has never been clearly established at the physiological or genetic level. In the present study, we used RNA-seq analysis to identify biofilm- and virulence-related genes displaying altered expression in biofilms compared to the planktonic condition. A flow cell system was employed to grow biofilms to obtain both structural and transcriptomic views of the biofilms. We found that 3615 genes were differentially expressed, confirming that biofilm and planktonic lifestyles are very different. As expected, the differentially expressed genes included those involved in biofilm formation, such as motility- and polysaccharide synthesis-related genes. The data show that quorum sensing is probably mediated by the AI-2/LuxO system in *V. tapetis* biofilms. The expression of genes encoding the Type VI Secretion System and associated exported proteins are strongly induced, suggesting that *V. tapetis* activates this virulence factor when living in biofilm.

## 1. Introduction

In their natural environment, most microbes live in surface-attached communities known as biofilms [1]. These sessile communities are embedded in a matrix of extracellular polymeric substances produced by the microorganisms themselves, which exhibit an altered physiological state and genetic profile [2]. The question of how gene expression differs in biofilms in comparison with planktonic cells has become a key research theme [3,4,5], given the evident link between biofilm lifestyle, and the persistence and virulence of many pathogens [6,7,8,9]. Successful host colonization depends on the activation and repression of numerous genes that lead to the formation of biofilms. In *Vibrio* species, the presence of mannose-sensitive haemagglutinin pili (MSHA), Type IV pili (TFP), and the flagellum are necessary for motility, allowing the exploration of surfaces. When a surface is recognized, cells attach onto it, and the subsequent loss of flagellum can serve as a signal to produce matrix substances [10]. The biofilm matrix is commonly composed of extracellular DNA (eDNA), proteins and extracellular polysaccharides [11]. Depending on the *Vibrio* species, different polysaccharides can be produced: VPS in *V. cholerae* [12], CPS in *V. parahaemolyticus* [13], or Syp in *V. fischeri* [14]. 

Quorum sensing (QS) communication has been shown to be involved in the regulation of biofilm formation [10]. Several QS systems have been discovered in *Vibrio* species since the first description of the LuxR/LuxI system of the symbiotic *V. fischeri* bacterium in the squid *Euprymna. scolopes* [15]. The most common *Vibrio* system, LuxS/LuxPQ, and also the CqsA/CqsS system, are known to regulate many virulence genes as well as biofilm formation in *V. fischeri*, *V. harveyi*, *V. anguillarum*, *V. cholerae*, and *V. vulnificus* [16,17,18]. All of the QS systems present in *Vibrio* involve the central activator LuxO, but the number of signals converging on LuxO differs [16].

Biofilms are often associated with chronic infections, as opposed to acute infection. RNA-seq studies in *V. vulnificus* and *V. cholerae* from planktonic cells grown in artificial seawater show the induction of genes involved in biofilm formation (genes for TFP, CPS, etc.). By contrast, when these bacteria are grown in human serum or in animal models, the induced genes were those involved in acute infections (genes for toxins, the type VI secretion system (T6SS), etc.) [19,20]. 

*Vibrio tapetis* is a marine Gram-negative bacterium responsible for diseases in fish and clam, and it is well-studied as the causal agent of Brown Ring Disease (BRD) in the clam *Ruditapes philippinarum*. Its genome was published in 2018, and genes related to host immunity resistance, secondary metabolites (siderophores, polyketide synthases, etc.), lipopolysaccharide O-antigen and the type IV secretion system (T4SS) were annotated [21]. BRD is a chronic shell disease characterized by a brown deposit of conchiolin on the inner surface of the clam shell. The attachment of *V. tapetis* onto the periostracal lamina on the inner surface of the shell is the initial event leading to BRD [22]. This bacterial attachment suggests that a biofilm is subsequently developed, as supported by a microscopic study of the Brown Ring syndrome that documented the presence of bacterial aggregates within the conchiolin deposit [23]. In vitro, *V. tapetis* forms various biofilm architectures with different matrix compositions, depending on the strain used [11]. One original feature is the presence, at the biofilm surface, of spherical components of a cellular nature containing DNA and proteins. These components likely originate from the bacterial cells to which they remain attached. To our knowledge, these components have so far only been described in *V. tapetis* biofilms [11]. 

To understand biofilm formation, mutants are generally constructed to decipher the involvement of the inactivated genes. Mutagenesis is quite difficult to perform in *V. tapetis*, because of a poor transformation efficiency that is probably due to the presence of several restriction systems in the genome (supplementary data [21]), and to date, only the *djlA* gene has been successfully knocked out [24]. This gene encodes a membrane-anchored DnaJ-Like protein that is involved in virulence. In view of this difficulty, we decided to use high-throughput RNA sequencing (RNA-seq) to identify the major transcriptomic features of *V. tapetis* CECT4600 biofilms compared to planktonic cells. Growing biofilms in flow cells under dynamic conditions (flow of culture medium) is the “gold standard” for the visualization of biofilms using confocal laser scanning microscopy (CLSM). This technique allows us to generate three-dimensional views for in-depth observations of biofilm structures, and to determine the biofilm parameters (biovolumes; average and maximal thicknesses). However, this technique is time-consuming, difficult to set up for large quantities of samples, and does not produce large amounts of biomass [25]. Therefore, biofilms for transcriptomic studies are usually grown using alternative methods yielding higher biomasses, but which do not provide complementary structural analyses of the biofilms. These methods include biofilm cultivation on glass beads [25], in microtiter plates (6- to 96-wells) [26,27,28,29], on immersed glass slides in batch cultures [30,31], or under dynamic conditions using a drip-flow system [32,33]. 

Here, we set up a new procedure to extract sufficient RNA from biofilms grown in a flow cell system to perform a transcriptomic study. This innovative set-up allows a genetic and microscopic investigation of the same biofilm, whereas most other studies use two independent set-ups to achieve the same objective. In addition to identifying genes involved in *V. tapetis* biofilm formation, we paid particular attention to virulence genes displaying an increased or decreased level of transcription in the biofilm, to obtain new insights into the biofilm–virulence relationship.

## 2. Results and Discussion

### 2.1. Transcriptional Activity in Biofilm Versus Planktonic Cells: Global Overview

**Figure 1:**
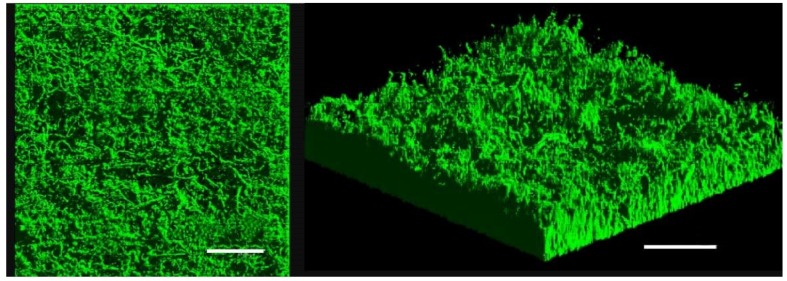
Confocal laser scanning microscopy (CLSM) observation of *V. tapetis* CECT4600-GFP biofilm grown for 48 h in a flow cell chamber at 18 °C, top view (left) and three-dimensional view (right), scale bar represents 50 µm.

Since BRD of the clam *R. philippinarum* involves *V. tapetis* biofilm formation on the inner surface of shells, we investigated the differences in gene expression between biofilms and planktonic cells. *V. tapetis* CECT4600-green fluorescent protein (GFP) biofilms were formed on glass slides in flow cells under a constant flow of culture medium [11]. *V. tapetis* formed non-structured (absence of mushroom-like structure) and rather homogeneous biofilms, with a biovolume reaching 16 µm^3^·µm^−2^ after 48 h of growth (Figure 1). Biofilms were grown for 48 h to obtain sufficient biomass to extract RNA, since the biovolume is known to be doubled after 48 h growth compared with 24 h [11].

We set up a procedure to harvest most of the bacteria from *V. tapetis* CECT4600 biofilms and lyse the bacteria that remained attached to the glass slide, as described in Section 3.5. Biofilms grown in three flow cell channels in parallel yielded 73.5 ± 2.3 µg of RNA. RNAs from planktonic cultures (free-living cells) were extracted during the stationary phase, yielding 80.5 ± 1.5 µg of RNA after 48 h of growth. The RNA quality was checked by measuring the OD_260_/OD_280_ ratio and also by the GATC laboratory (Eurofins, GATC Biotech, Konstanz, Germany) before sequencing. An average of ≈ 8 million reads were obtained per sequenced sample. About 97% of total reads were mapped to the annotated *V. tapetis* CECT4600 genome (GenBank assembly accession number GCA_900233005.1) (Supplementary Table S1). Deviations in the RNA-seq analysis can be due to differences in the gene lengths and to the size of the library for each sample. Normalization was performed using the variance analysis package DEseq2 [34,35], followed by the calculation of the *p*-value to determine the statistical significance in the number of reads per gene among the biological samples. A *p*-value adjustment was performed to take multiple testing into account, and to check the false discovery rate (FDR) at a chosen level α. For this analysis, a BH *p*-value adjustment was performed, and the level of controlled false positive rate was set at 0.05 [36]. 

**Figure 2:**
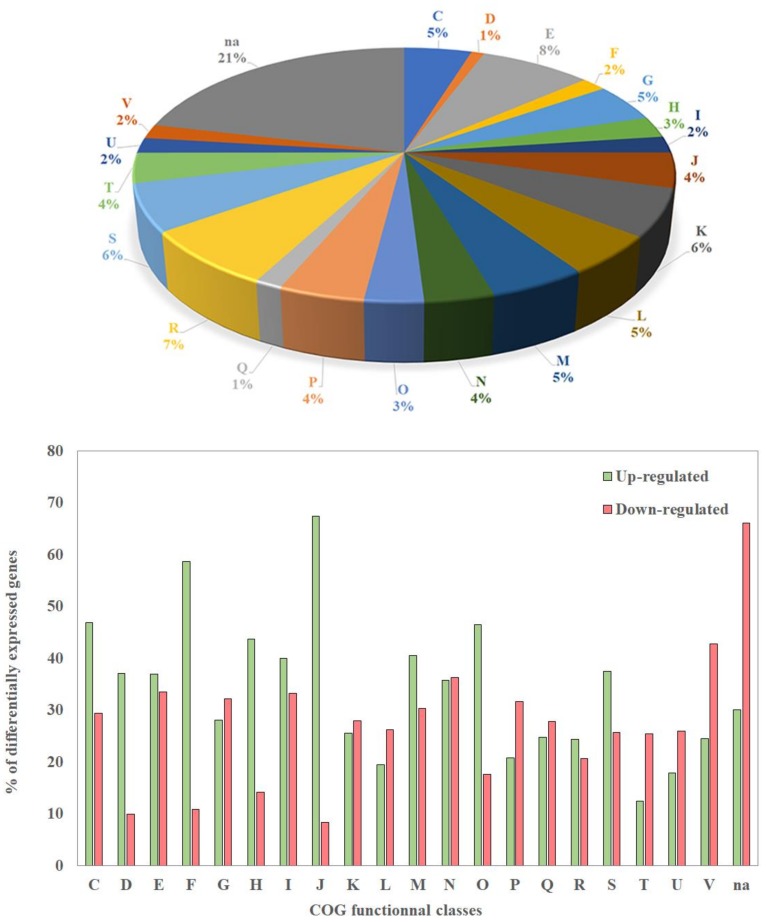
Distribution of differentially expressed genes in clusters of orthologous groups (COG) classes (top panel) and percentage of genes of each COG which are up- or down-regulated in the biofilm vs planktonic condition (bottom panel). COG classes; C: Energy production and conversion. D: Cell cycle control, cell division, chromosome partitioning. E: Amino acid transport and metabolism. F: Nucleotide transport and metabolism. G: Carbohydrate transport and metabolism. H: Coenzyme transport and metabolism. I: Lipid transport and metabolism. J: Translation, ribosomal structure and biogenesis. K: Transcription. L: Replication, recombination and repair. M: Cell wall/membrane/envelope biogenesis. N: Cell motility. O: Posttranslational modification, protein turnover, chaperones. P: Inorganic ion transport and metabolism. Q: Secondary metabolites biosynthesis, transport and catabolism. R: General function prediction only. S: Function unknown. T: Signal transduction mechanisms. U: Intracellular trafficking, secretion, and vesicular transport. V: Defense mechanisms. Na: Not assigned.

A total of 3615 genes (62%) were significantly differentially expressed in the biofilm versus the planktonic condition (FDR < 0.05). A total amount of 1816 (30%) and 1832 (32%) genes were up- and down-regulated in the biofilm condition, respectively (Supplementary Table S2). Due to the large number of differentially expressed genes (DEGs), we present here a first overview of the functional classes of their products, using a classification based on clusters of orthologous groups (COG) (Figure 2 top panel). 

Although DEGs belong to 20 COGs, the largest proportion of these genes (≈ 21%) encoded proteins that were not assigned to a specific COG (Figure 2, top panel). The largest proportions of DEGs assigned to COGs were found in classes R and E (general function prediction only; amino acid transport and metabolism), which contain about 7 and 8% of the DEGs, respectively (Figure 2, top panel), but the percentages of up-and down-regulated genes inside each of these two COGs were similar (Figure 2 bottom panel). Among the genes involved in translation, ribosomal structure, and biogenesis (class J), as well as the genes involved in nucleotide transport and metabolism (class F), 60 to 70% were up-regulated, whereas only 10% were down-regulated in the biofilm. Related DEGs belonging to COG classes C, D, H, I, M, and O, and S were moderately up-regulated, but the differences in the percentages of up- and down-regulated genes in each class were smaller than for classes J and F (40 to 60%) (Figure 2 bottom panel). On the contrary, a large proportion of DEGs involved in signal transduction mechanisms (class T), as well as in intracellular trafficking, secretion and vesicular transport (class U), were mostly down-regulated (Figure 2 bottom panel). Finally, no DEG belonging to COG classes A, B, Y, and Z was found, as expected, since these COGs concern eukaryotic cells. These results showed that many genes involved in general bioprocesses or in specific functions display a transcription variation. This is not surprising, since the physiology of cells in biofilms is quite different compared to those observed in planktonic bacteria. 

**Figure 3:**
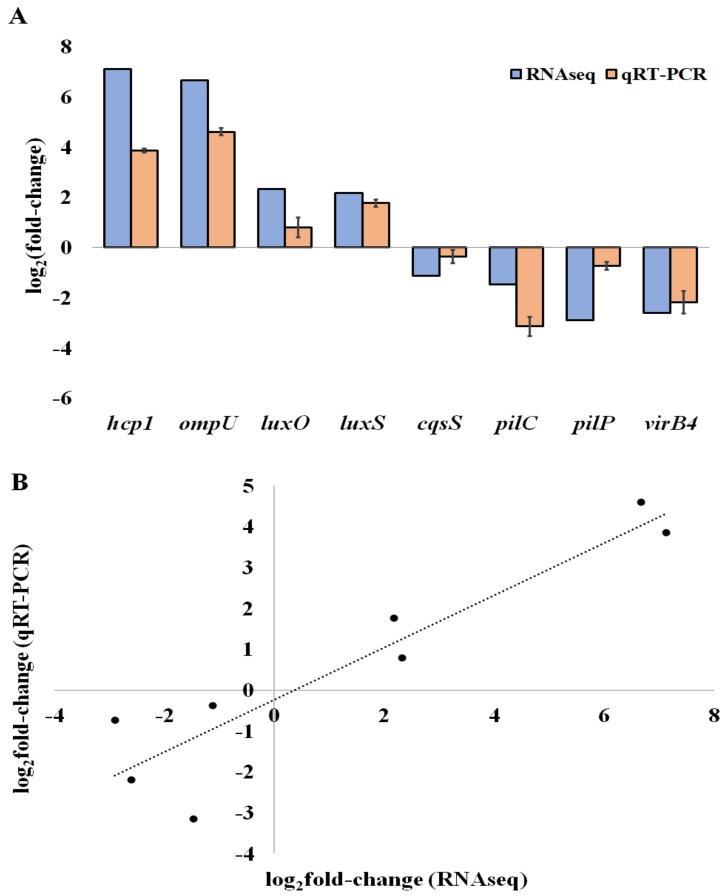
QRT-PCR validation of differentially expressed genes. (**A**) The log_2_ of the fold-change (Log_2_FC) expression of genes in *V. tapetis* CECT4600 biofilm cells compared to planktonic cells. (**B**) Correlation of Log_2_FC of the expression levels of genes determined by RT-qPCR and by RNA-seq. For RT-qPCR experiments, the bars represent the mean, and the error bars the standard error of the mean (mean ± SEM).

To corroborate our RNA-seq results, we first selected eight genes that were differentially expressed in the biofilms: three are related to QS (*luxO, luxS* and *CqsS*), two to motility and attachment (*pilC, pilP*), and three to virulence (*hcp1, ompU*, and *virB4*). Then, we assessed their relative expression levels by qRT-PCR (Figure 3A). We found that the gene expression data of the eight selected DEGs determined by RNA-seq correlates with the levels measured by RT-qPCR, with a high Pearson correlation coefficient (0.92), leading to the validation of the RNA-seq results (Figure 3B). 

To validate our experimental set-up, we focused our RNA-seq analysis on genes that are involved in different phenomena occurring in the course of biofilm formation: attachment and motility, matrix production, and QS. We searched for such genes in the *V. tapetis* genome, and then analysed their relative expression level in biofilm compared to free planktonic cells, using RNA-seq. Since only microscopic observations are so far available concerning the formation of *V. tapetis* biofilms, our transcriptomic study should therefore contribute to the understanding this phenomenon at the molecular level. Then, to investigate the link between the biofilm and virulence, we performed the same analyses on genes involved in virulence.

### 2.2. Attachment and Motility Involving TFP

At least two sets of TFP genes can be found in *Vibrio* species: chitin-regulated pilus (ChiRP) genes including *pilA*, and mannose-sensitive haemagglutinin (MSH) genes including *mshA*. Both of these pili biogenesis genes are involved in biofilm formation. In *V. parahaemolyticus*, MSH pili play a major role in the environmental condition and the aggregation of cells to each other during biofilm formation, leading to the formation of microcolonies [37]. ChiRP pili are more involved in the first step of colonization of the surface by *V. parahaemolyticus* [37]. ChiRP pili are also involved in twitching, which is a type of surface motility that occurs through the binding of a pilus to a fixed surface, followed by retraction of the pilus. This motility type is widely encountered in bacteria, and has been described and modelled in *V. cholerae* [38]. In the *V. tapetis* genome, we identified one gene cluster involved in MSH (a2701 to a2718) assembly, and two clusters involved in ChiRP (a2965 to a2969 and a0250 to a0253) assembly. RNA-seq analyses revealed a two-fold induction of the two *mshA* genes (a2706 and a2708), and of the gene encoding the MSHA pilin (a2707) (Table 1), suggesting that MSHA pili are also involved in biofilm formation in *V. tapetis*. 

By contrast, ChiRP genes, including *pilC* and *pilMNOPQ*, were down-regulated in biofilm between 1.7 and 3.09-fold in RNA-seq analyses, and *pilC* and *pilP* were confirmed as being down-regulated by qRT-PCR, with relative expressions decreasing 8- and 1.6-fold, respectively (Figure 3). We previously observed that *V. tapetis* CECT4600 cells attached to the glass surface do not display a surface motility behaviour, such as swarming or twitching (see movie in Supplementary Data, derived from [11]). This observation is consistent with the decrease of ChiRP gene transcription, assuming that the ChiRP pili are responsible for twitching motility in *V. tapetis*, as observed in *V. parahaemolyticus*. 

### 2.3. Matrix Production-Associated Genes

Production of mature biofilms requires extracellular matrix components that hold the cells together and that keep the biofilm attached to the surface. We previously showed that the biofilm matrix of *V. tapetis* includes β-polysaccharides [11]. The capsular polysaccharide (CPS) or exopolysaccharide (EPS or VPS in *V. cholerae*) loci involved in biofilm formation have been identified from numerous *Vibrio* species. In *V. cholerae*, VPS is the main compound found in the matrix (up to 50%). In *V. tapetis*, we found five genes potentially involved in polysaccharide biosynthesis, and probably part of a *vps* or *cps*-like locus (b1177 to b1181). Our RNA-seq analyses revealed that these genes were all down-regulated in biofilm condition (Table 2). However, we also found four genes of the *eps* cluster (a3108 to a3110) and four genes of the *gsp* cluster (a3115 to a3119) that are induced in *V. tapetis* biofilm formation, compared to free-living cells (Table 2).

The *galU* (a2724) and *galE* (a2985) genes were found in the *V. tapetis* genome; these two genes are respectively involved in UDP-glucose and UDP-galactose synthesis; both are necessary for biofilm production by *V. cholerae* [10]. The transcription of *galU* and *galE* was respectively increased 2.7 and 1.6-fold in *V. tapetis* biofilm, in agreement with their potential involvement in matrix exopolysaccharide synthesis.

In *V. fischeri*, biofilm formation depends on a cluster of 18 polysaccharide biogenesis genes (denoted as *syp* for symbiosis polysaccharides) [14]. This locus is lacking in *V. cholerae*, but it is conserved in other pathogenic *Vibrio* sp., such as *V. parahaemolyticus* and *V. vulnificus. syp* genes are essential for the establishment of a symbiosis between *V. fischeri* and the squid *Euprymna scolopes*. The products of these genes have been suspected to be involved in early stages of biofilm formation, where SypG is the main positive regulator and where SypE is a second positive regulator [14]. *sypG* is regulated at the transcriptional level by the sensor kinase RscS [39]. In the *V. tapetis* CECT4600 genome, 19 *syp* genes were identified (Supplementary Table S3) that correspond to the whole clusters found in the three other *Vibrio* sp. mentioned above, except for the *sypE* gene, which is lacking. No protein or nucleotide sequences could be found corresponding to RscS or *rscS*, suggesting that *sypG* is activated by another sensor. The RNA-seq results presented here showed that *sypG* (initially annotated *luxO* in *V. tapetis*) is up-regulated 1.5-fold in our biofilm condition, indicating that it plays a role in biofilm formation. Surprisingly, all the other *syp* genes, which are directly involved in polysaccharide biosynthesis and transport, were inhibited (Supplementary Table S3). Yip et al. suggested that environmental conditions probably play a predominant role to reproduce biofilm formation and polysaccharide biosynthesis [14]. These authors showed that only an overexpression of *sypG* was able to activate *syp* gene transcription. The experimental conditions used in the present study are probably not close enough to the required environmental conditions, which implies that *sypG* expression was insufficient to induce the other *syp* genes.

### 2.4. QS Associated Genes

Among the existing QS systems discovered in *Vibrio* species [16], we were able to identify *cqsA/cqsS* and *luxS/luxPQ* in the *V. tapetis* genome. CqsA and LuxS are involved in the production of the autoinducers CAI-1 and AI-2, respectively, with CqsS and LuxP/Q corresponding to their respective sensors, which enhance the subsequent phosphorylation of LuxU and then LuxO. In the biofilm condition used here, *cqsS* expression was inhibited compared to the planktonic condition (Table 3), its transcription decreased 2.1-fold and 1.2-fold using RNA-seq and qRT-PCR, respectively. *cqsA* was not differentially expressed in the RNA-seq data, and none of the tested qPCR primers was able to amplify its complementary DNA (cDNA). In the case of *V. tapetis*, the other gene identified involved in autoinducer production is *luxS*; the induced fold change for this gene was 4.5 and 3.44, as estimated by RNA-seq and qRT-PCR assays, respectively (Table 3 and Figure 3). *luxQ* encodes a subunit of the LuxP/Q AI2 sensor; this gene was down-regulated in biofilm (about 1.4-fold), whereas *luxP* was not differentially expressed under our experimental conditions (Table 3). In most *Vibrio* species, the different QS systems converge to the central regulator LuxO [40]. Our data showed that *luxO* was up-regulated in the biofilm (5-fold) (Table 3), suggesting that LuxS is involved in the biofilm control of *V. tapetis* via the central regulator LuxO. 

The fact that *luxP/Q* were not induced can probably be explained by the concentration of their products in the membrane, which would be sufficient to transfer the signal to the LuxO intracellular regulator. LuxP/Q are indeed constitutively present and organized as a complex in the inner membrane of the cell, and the AI-2 concentration is the main parameter allowing a switch between phosphatase (low AI-2 concentration) and kinase activities (high AI-2 concentration) [41].

### 2.5. Virulence-Associated Genes

Virulence is often described as a balance between chronic infection (biofilm) and acute infection (free cells). Many studies have shown an opposite regulation of the two mechanisms, suggesting that cells are able to wait for the right opportunity to move from biofilm to free living cells, and vice-versa [42,43]. BRD probably requires both virulence and biofilm genes. Dias et al. analysed the genome of 17 *V. tapetis* strains, including the CECT4600 strain, focusing on the T4SS gene cluster (*virB*), since this latter has only been identified in the genomes of the 13 *V. tapetis* strains virulent against bivalve molluscs [21]. In the present study, RNA-seq analysis showed that eight genes of the T4SS cluster were down-regulated in the biofilm condition (Supplementary Table S4). We validated this observation by qRT-PCR analysis of the *virB4* mRNA level, which was 5-fold lower in the biofilm compared to the planktonic condition (Figure 3). This result suggested that the biofilm lifestyle represses the T4SS in *V. tapetis*.

So far, only the *djlA* gene has been successfully knocked out in *V. tapetis*. DjlA is a membrane-anchored DnaJ-like protein, and its mutant is unable to induce BRD and kill the *R. philippinarum* clam [24]. Compared to other similar proteins, DjlA was suspected to be involved in the assembly of system(s) responsible for secreting cytotoxic factors [24]. In our study, no variation was observed in the transcriptional activity of *djlA*, suggesting that this virulence factor is not over-produced in biofilm. 

It is not easy to use a global approach to analyse genes involved in virulence, since the latter is dependent on direct and indirect mechanisms. By comparing the *V. tapetis* CECT4600 genome with all *Vibrio* genomes available on the MAGE platform and using the virulome tool (www.genoscope.cns.fr), we identified five other genes of *V. tapetis* CECT4600 that are likely to be involved in virulence (*luxS, a0599, ompU, apxlB, b1661* and *cqsA* (Supplementary Table S5)). The expression levels of *luxS* and *cqsA*, which are involved in QS, were discussed above in Section 2.4. Among the four other genes, *a0599* was not differentially expressed, but the three others were altered. RNA-seq revealed a 2.4-fold induction of *apxlB*, a gene which is involved in repeats-in-toxin (RTX) toxin translocation. *b1661*, a gene similar to the gene encoding a thermolabile hemolysin in *V. parahaemolyticus*, showed a 2.5-fold down-regulation. 

*OmpU* is a gene encoding an outer membrane protein, which is involved in the adherence of *V. vulnificus* and *V. mimicus* to host cells [44,45]. Secretome analysis of *V. tapetis* has suggested the involvement of OmpU in the bacterial virulence [46]. RNA-seq analysis showed that its expression was induced 101-fold in the biofilm condition, representing the third most strongly induced gene in biofilm (Table 4). This trend was confirmed by qRT-PCR with an induction factor of 27.75 ± 3.79. Depending on the *Vibrio* species, OmpU may have different levels of involvement in biofilm formation. Cai et al. showed that the decrease of OmpU production impaired biofilm formation of *V. alginolyticus* [47], whereas OmpU appears to limit it in *V. anguillarum* [48]. In the present study, the strong up-regulation of its gene suggests that OmpU plays an important role in *V. tapetis* biofilm formation and/or maintenance. 

The RNA-seq data revealed that six genes displayed an induction fold-change value higher than 50 in biofilms (Table 4). In addition to *ompU* discussed above, three of these genes are involved in virulence: *b0571, b0572*, and *b0573*. The highest induction factor observed in the biofilm condition was obtained for the *b0573* gene (138.9-fold) (Table 4), and the up-regulation was confirmed by qRT-PCR, albeit with a lower induction factor (14.51 ± 1.42) (Figure 3). The product of this gene is predicted to be the T6SS component Hcp (hemolysin coregulated protein) which exhibits a close similarity (93%) with the Hcp-3 protein of the fish pathogen *Aliivibrio wadonis*. Two other genes in the T6SS cluster were also highly up-regulated in biofilm (Table 1): *b0571* (62-fold), and *b0572* (90-fold) encoding the TssB/TssC complex, two proteins of the T6SS contractile sheath. 

In the genetic environment of *V. tapetis b0571-3*, we identified a cluster of 19 genes (*b0557* to *b0575*) potentially involved in the biogenesis of a T6SS (Table S6). T6SS is a complex multicomponent secretion machine that is often involved in interactions with eukaryotic hosts, or in a pathogenic or a symbiotic relationship. The T6SS gene cluster usually encodes between 12 and 25 proteins [49]. In our transcriptome analysis, the 19 T6SS genes (*b0557* to *b0575*) were over-expressed from 1.9-fold to 138.9-fold (Table S6), meaning that the entire T6SS gene cluster is up-regulated in the biofilm condition. 

T6SS components are encoded by gene clusters that vary in their organization. These clusters were initially named IAHP (for IcmF-associated homologous proteins) because they contain a gene encoding an IcmF-like component [50]. In 2006, the *V. cholerae* IAHP cluster was identified as a new T6SS cluster and the genes were named *vas* for virulence-associated secretion [51]. Hcp and VgrG are two proteins secreted through T6SS. In *V. cholerae*, these two proteins are involved in the cytotoxicity against the amoeba *Dictyostelium discoideum* [51]. In *V. tapetis* biofilm, we showed that the two genes encoding Hcp (*b0573*) and VgrG (*b0575*) proteins were up-regulated (Supplementary Table S6). In 2016, Linares et al. also observed that Hcp1 is over-produced by *Shewanella frigidimarina* NCIMB400 during early biofilm formation [52]. 

In our biofilm condition, *icmF* was induced 2-fold and *clpV* 3-fold, while *vasA* and *vasB* were up-regulated 5.3 and 3.7-fold, respectively. 

Taken together, these results suggested that the T6SS genes and the two proteins Hcp and VrG potentially involved in the virulence of *V. tapetis* are strongly expressed during biofilm formation, and could therefore be preponderant actors in BRD.

## 3. Materials and Methods

### 3.1. Bacterial Strains

The reference strain CECT4600 of *V. tapetis* used in this study for the RNA-seq analysis was isolated from the venerid clam *R. philippinarum*, affected by BRD [24]. *V. tapetis* CECT4600-GFP (carrying the pVSV102 plasmid; *gfp*, Km^R^) was used only for CLSM biofilm observations [11]. *V. tapetis* was grown in Zobell medium (4 g/L tryptone; 1 g/L yeast extract; sea salts (Sigma-Aldrich, Saint-Louis, MO, USA), 30) at 18 °C. Kanamycin (Km) was used at 100 µg/mL during the cultivation of *V. tapetis* CECT4600-GFP.

### 3.2. Planktonic Growth

Zobell medium (10 mL) was inoculated at an OD_600_ = 0.05 with an overnight culture, and incubated at 18 °C for 48 h with vigorous agitation. Then, 1 mL of planktonic cells were harvested by centrifugation (10 min, 4000 g at 4 °C), and the pellet was used for RNA extraction.

### 3.3. Biofilm Culture

*V. tapetis* biofilms were grown at 18 °C under hydrodynamic conditions in a three-channel flow cell (1 × 4 × 44 mm; Biocentrum, DTU, Denmark). The flow system was assembled, prepared, and sterilized as described previously [53]. The substratum consisted of a microscope glass coverslip (24 × 50 st1 (KnittelGlasser, Braunschweig, Germany)). Each channel was inoculated with 250 μL of an overnight culture of *V. tapetis* diluted to an OD_600_ = 0.5 in artificial sea water (ASW: 30 g/L sea salts (Sigma-Aldrich, Saint-Louis, MO)). An attachment step was performed for 2 h without any flow of ASW or medium. A flow of Zobell medium was then applied at a rate of 2.5 mL/h for 48 h, using a Watson Marlow 205 U peristaltic pump (Watson Marlow, Falmouth, UK). Biofilms were then observed by CLSM as described below.

### 3.4. Confocal Laser Scanning Microscopy (CLSM)

Biofilms formed by *V. tapetis* CECT4600-GFP were observed by monitoring the GFP fluorescence with a TCS-SP2 microscope (Leica Microsystems, Heidelberg, Germany), using a 63× oil immersion objective. GFP was excited at 488 nm and fluorescence emission was detected at between 500 and 550 nm. Images were acquired at intervals of 1 µm throughout the whole depth of the biofilm. Leica LAS AF software (Leica Microsystems, Heidelberg, Germany) was used for visualization and processing of three-dimensional (3D) image data (volume rendering with shadow projection). Quantitative analyses of image stacks were performed using the COMSTAT software (http://www.imageanalysis.dk/) [54]. At least three image stacks from each of three independent experiments (nine stacks in total) were used for each analysis.

### 3.5. RNA Extraction

Total RNA extractions were performed by using the MasterPure Complete RNA purification Kit (Epicentre, Illumina, Madison, USA). For each condition (biofilm or planktonic), RNA extractions were carried out from three independent experiments. For planktonic cells, the extraction was carried out according to the supplier’s protocol. For biofilms, we set up a procedure to harvest the bacteria from flow cell-grown biofilms. For a single RNA extraction, *V. tapetis* CECT4600 biofilms were grown in three flow cell channels in parallel. After 48 h of biofilm growth in Zobell medium at a flow rate of 2.5 mL·h^−1^, the flow was progressively increased to 25 mL·h^−1^, and maintained at that value for 5 min, to ensure that all planktonic cells were removed from the channels. Then, the flow was stopped, the tubes at the end of the flow cell were cut, and their contents were recovered by manually flushing each channel aseptically with a syringe. A total of 300 µL of tissue and cell lysis solution (Epicentre) were introduced into each channel to release the remaining attached cells. The contents of the three channels were pooled. The following steps of the RNA extraction were performed according to the supplier’s protocol. The glass slides were observed after the extraction step to check for the absence of any remaining biofilm, indicating adequate efficiency of the cell lysis. The amount and quality of RNA were assessed using a NanoPhotometer N60 (Implen) spectrophotometer. The RNA integrity number (RIN) was checked using an Agilent 2100 Bioanalyzer 2100 (Agilent Technologies), yielding a value ≥8. Total RNAs were then stored at −80 °C.

### 3.6. RNA-Sequencing and Data Analyis

Ribosomal RNA depletion, cDNA library preparation, and Illumina sequencing were performed at the GATC laboratory (Eurofins, GATC Biotech, Konstanz, Germany). Raw data analysis and differential expression evaluation were carried out using the ABiMS Galaxy platform, Station Biologique de Roscoff, CNRS/Sorbone Université, France (http://galaxy3.sb-roscoff.fr/). Preliminary quality control analysis of the fastq files was carried out with the tool FastQC v0.7.0 [55], and sequences were then trimmed using Trimmomatic v0.36.4 [56]. RNA-seq reads were mapped to the reference genome of *V. tapetis* CECT4600 strain (GenBank assembly accession number GCA_900233005.1) with the Boowtie2 software [57]. HTseq-count v0.6.1 using the union model was used to calculate the number of reads mapping to each feature [58]. The statistical analysis of differentially expressed genes was performed with the SARTools R package, including the DESeq2 package [34,35,59]. The analysis process included data normalization and tests for differential expression for each feature between the conditions and raw *p*-value adjustment. A *p*-value ≤ 0.05 is considered as statistically significant. Genes are considered as significantly differentially expressed if the *p*-value adjusted (*p*adj) by FDR (false discovery rate) is <0.05 [36] and the log2fold-change (log2FC) of the gene expression in the biofilm versus the planktonic condition is positive (up-regulated) or negative (down-regulated). The sequencing data obtained in this study have been deposited in NCBI Gene Expression Omnibus [60], and are accessible through GEO Series accession number (GSE120499).

### 3.7. mRNA Quantification by Real-Time Reverse Transcription PCR

The RNA-seq results were validated using qRT-PCR. Total RNAs were converted to cDNAs using the High Capacity cDNA Archive Kit (Applied Biosystems, Foster City, CA, USA). Primers were designed using the Primer Express software (Applied Biosystems), purchased from Eurogentec (Liege, Belgium) and are listed in Supplementary Table S7. Real-time PCR was performed using a 7300 Real Time PCR System apparatus (Applied Biosystems) and SYBR Green PCR Master Mix (Applied Biosystems), using the procedures previously described [61]. Transcripts levels were obtained by the comparative Ct (2^−ΔΔCT^) method using 16S ribosomal RNA (rRNA) as an endogenous control [61].

## 4. Conclusions

The novelty of our approach stems from the transcriptomic analysis of a biofilm grown under dynamic conditions, combined with observation of the biofilm by confocal laser scanning microscopy. This contrasts with most other studies, which use two independent set ups. As expected, the expression of many genes was altered, confirming that biofilm and planktonic lifestyles are very different. Our analyses focused on genes related to biofilm formation and virulence, showing that motility was abolished in biofilm and that QS mainly occurred via the LuxS pathway. We found that T6SS was greatly induced, suggesting that this system might be involved in *V. tapetis* colonization and the formation of biofilm on conchiolin secretions in the case of BRD in clams.

## Figures and Tables

**Table 1:** Genes involved in attachment and motility.

Gene ID	Gene Name	FC	Log_2_FC	Product
a2706	*mshA*	1.9	1	Mannose-sensitive haemagglutinin A
a2707		2.1	1.1	MSHA pilin protein MshA
a2708	*mshA*	1.9	0.9	Mannose-sensitive haemagglutinin A
a0251	*pilC*	0.3	−1.5	Type IV pilin assembly protein PilC
a2965	*pilQ*	0.2	−2.2	Fimbrial assembly protein PilQ (fragment)
a2966	*pilP*	0.1	−2.9	Putative pilus assembly protein PilP
a2967	*pilO*	0.3	−1.6	Putative pilus assembly protein PilO
a2968	*pilN*	0.2	−2.3	Putative TFP pilus assembly protein PilN
a2969	*pilM*	0.4	−1.4	Putative Type IV assembly protein PilM

FC: fold change.

**Table 2:** Genes involved in polysaccharide production (with KEGG:KO).

Gene ID	KO	Gene Name	FC	Log2FC	Product
*b1181*	K03606	*wcaJ*	0.1	−2.8	Putative UDP-sugar lipid carrier transferase
*b1180*	K20920		0.3	−1.7	Polysaccharides biosynthesis/export protein VpsM
*b1179*	K20988		0.4	−1.2	Polysaccharides biosynthesis/export protein VpsN
*b1178*	K16554		0.2	−1.9	Polysaccharide biosynthesis transport protein
*b1177*	na		0.2	−1.9	Putative polysaccharide biosynthesis protein
*a3110*	K02461	*epsL*	1.9	0.9	Type II Secretion System protein L
*a3109*	K02462	*epsM*	1.5	0.5	Type II Secretion System protein M
*a3108*	K02463	*epsN*	1.4	0.4	Type II Secretion System protein N
*a3119*	K02452	*epsC*	1.6	0.7	Type II Secretion System protein C
*a3118*	K02453	*gspD*	2.1	1	General secretion pathway protein D
*a3117*	K02454	*gspE*	2.2	1.2	General secretion pathway protein E
*a3116*	K02455	*gspF*	2	1	General secretion pathway protein F
*a3115*	K02456	*gspG*	2.7	1.4	General secretion pathway protein G
*a2724*	K00963	*galU*	2.7	1.4	UTP-glucose-1-phosphate uridylyltransferase
*a2985*	na	*galE*	1.6	0.7	UDP-glucose 4-epimerase

KO: KEGG functional orthologues, **na:** no KO assigned.

**Table 3:** Quorum sensing-associated genes.

Gene ID	Gene Name	FC	Log2FC	Product
*b1775*	*cqsS*	0.4	−1.1	CAI-1 autoinducer sensor kinase/phosphatase CqsS
*b1774*	*cqsA*	Not DEG		CAI-1 autoinducer synthase
*a0117*	*luxS*	4.5	2.2	S-ribosylhomocysteinase
*b0125*	*luxQ*	0.7	−0.5	Autoinducer 2 sensor kinase/phosphatase LuxQ
*b0126*	*luxP*	Not DEG		Autoinducer 2-binding periplasmic protein LuxP
*a1225*	*luxO*	4.9	2.3	Regulatory protein LuxO

Not DEG: not differentially expressed genes.

**Table 4:** Genes with induction fold-change >50 in biofilm.

Gene ID	Gene Name	FC	Log_2_FC	Product
*b0573*		138.9	7.1	T6SS component Hcp
*a2618*	*yfiD*	116.3	6.8	Pyruvate formate lyase subunit
*a1598*	*ompU*	101.1	6.6	Outer membrane protein U
*b0572*		90	6.5	T6SS component TssB (ImpB/VipA)
*b0571*		62.6	5.9	T6SS component TssC (ImpC/VipB)
*a3240*		55.9	5.8	Protein of unknown function

## Supplementary Materials

The following are available online at http://www.mdpi.com/2076-0817/7/4/92/s1, Table S1: Summary of RNA-seq and mapping results, Table S2: Summary of statistical analysis with DESeq2, Table S3: *syp* genes cluster in *V. tapetis*, Table S4: List of T4SS genes down-regulated in biofilm condition, Table S5: Genes identified in the virulome of *V. tapetis* CECT4600 using all *Vibrio* genomes available in the MAGE platform, Table S6: Genes involved in the Type VI Secretion System. Table S7: Primers used in qRT-PCR.
