# Supplementary material for: Relation between Biofilm and Virulence in Vibrio tapetis: A Transcriptomic Study
Source: Pathogens. 2018 Nov 26;7(4):92. doi: 10.3390/pathogens7040092 (PMC6313714; doi:10.3390/pathogens7040092)
Supplement: Supplementary file 1 [file pathogens-07-00092-s001.pdf]

Table S1: Summary of RNA-seq and mapping results.

Condition	Millions of reads	% reads aligned to genome
**Biofilm 1**	8.4	97.10
**Biofilm 2**	7.8	97.31
**Biofilm 3**	7.4	97.10
**Planktonic 1**	9.7	97.19
**Planktonic 2**	10.8	96.00
**Planktonic 3**	4.8	96.53

Table S2: Summary of statistical analysis with DESeq2.

	Nbr of features	UP	DOWN
**Total in genome**	5780	/	/
**Total RNAseq detected**	5480	/	/
**Total DEG**	3693	1846	1847
**CDS, fCDS DEG**	3615	1816	1832

DEG: Differentially expressed genes.

Table S3: ***syp*** **genes cluster in** ***V. tapetis.***

Gene ID	Gene name	FC	Log2FC	function
b1501		0.3	-1.5	Sugar transferase SypR involved in lipopolysaccharide synthesis
b1502		0.2	-2.	Glycosyltransferase SypQ
b1503		0.2	-2.5	Glycosyltransferase SypP
b1504		0.06	-4	Hypothetical protein
b1505		0.2	-2.2	Polysaccharide biosynthesis chain length regulator SypO
b1506		0.3	-1.7	Glycosyltransferase SypN
b1507		0.1	-2.9	Acethyltransferase SypM
b1508		0.1	-3.1	Membrane protein SypL involved in exopolysaccharide production
b1509		0.1	-3.6	Membrane protein SypL involved in exopolysaccharide production
b1510		0.2	-2.3	Oligosaccharide translocase SypK
b1511		0.2	-2.3	Glycosyltransferase SypJ
b1513		0.26	-1.9	Glycosyltransferase SypI
b1514	*sypH*	0.7	-0.6	Glycosyltransferase SypH
b1515	*sypG*	1.5	0.6	Sigma-54 dependent transcriptional regulator SypG
b1516		0.4	-1.3	Signal transduction histidine kinase SypF
b1517		0.3	-1.8	Predicted protein SypD
b1518		0.2	-2.3	Periplasmic protein SypC involved in polysaccharide export
b1519		0.2	-2.1	Outer membrane protein SypB
b1520		0.1	-2.8	Anti-anti-sigma regulatory factor SypA

Table S4: List of T4SS genes down-regulated in biofilm condition.

Gene ID	Gene name	FC	Log2FC	Product
a3553	*virB2*	0.3	-1.6	Putative type IV secretion system protein B2
a3552	*virB3*	0.3	-1.7	Putative type IV secretion system protein B3
a3554	*virB4*	0.1	-2.6	Putative type IV secretion system protein B4
a3557	*virB6*	0.2	-2.3	Putative type IV secretion system protein B6
a3558	*virB8*	0.2	-2.4	Putative type IV secretion system protein B8
a3559	*virB9*	0.2	-2.5	Putative type IV secretion system protein B9
a3560	*virB10*	0.14	-2.8	Putative type IV secretion system protein B10
a3565	*virB11*	0.2	-3.9	Putative type IV secretion system protein B11

Table S5: Genes identified in virulome of *V. tapetis* CECT4600 using all *Vibrio* genomes available in the MAGE platform.

Gene	Product	Organism	Function	*V. tapetis* CECT4600	
				Gene id	Log2FC
luxS	S-ribosylhomocysteinase	*V. cholerae*	1	*a0117*	2.2
a0599	putative Paraquat-inducible protein B	*V. parahaemolyticus*	2	*a0599*	not DEG
ompU	Outer membrane protein U	*V. vulnificus*	2	*a1598*	6.6
apxIB	Toxin RTX-I translocation ATP-binding protein	*V. cholerae*	3	*b0152*	-1.3
b1661	Thermolabile hemolysin	*V. parahaemolyticus*	3	*b1661*	-1.3
cqsA	CAI-1 autoinducer synthase	*V. cholerae*	1	*b1774*	not DEG

Functions: 1. Regulation of virulence-associated genes, Regulation, Quorum sensing. 2. Offensive virulence factors, Adherence.3. Secreted proteins. Not DEG: not differentially expressed gene (*p*adj>0.05).

Table S6: Genes involved in Type VI Secretion System.

Gene ID	Gene name	FC	Log_2FC	Product
b0557	*icmF*	2	1	T6SS component TssM (IcmF/VasK)
b0558		2.1	1	T6SS outer membrane component TssL (ImpK/VasF), DotU family
b0559		3	1.5	T6SS component TssK (ImpJ/VasE)
b0560		3	1.5	T6SS secretion lipoprotein TssJ (VasD)
b0561		3.6	1.8	T6SS FHA domain protein ImpI/VasC
b0562		2	1	Hypothetical protein
b0563		2.4	1.2	Hypothetical protein
b0564		1.9	1	Hypothetical protein
b0565		3.2	1.7	D-ala D-ala ligase and related ATP-grasp enzymes-like
b0566	*clpV*	3	1.6	T6SS chaperone ClpV (TssH)
b0567	*vasB*	3.7	1.9	T6SS component TssG (ImpH/VasB)
b0568	*vasA*	5.3	2.4	T6SS component TssF (ImpG/VasA)
b0569		4.3	2.1	T6SS lysozyme-like component TssE
b0570		5.1	2.3	Putative T6SS protein VasRB-1
b0571		62.6	6	T6SS component TssC (ImpC/VipB)
b0572		90	6.5	T6SS component TssB (ImpB/VipA)
b0573		138.9	7.1	T6SS component Hcp
b0574		20	4.3	T6SS component TssA (ImpA)
b0575		3	1.6	VgrG protein

Table S7: Primers used in qRT-PCR. Primer were designed with Primer Express software.

Primer	Sequence (5′→3′)	Amplified gene (ID)
***luxO*F**	GAG-CGT-GTT-CAT-TTG-CGG-A	*luxO* (*a1225*)
***luxO*R**	TCC-GCG-CAG-ACT-TCT-TTA-CC	
***pilC*F**	AAC-GCC-GCA-GTG-TTT-CTT-TT	*pilC* (*a0251*)
***pilC*R**	TCG-CAC-TGA-CTT-TGT-TGG-AGA	
***16S*F**	TTT-GCC-AGC-GAG-TAA-TGT-CG	*16srRNA 2*
***16S*R**	GGT-TTA-TCA-CCG-GCA-GTC-TCC	
***cqsS*F**	TGG-CGG-GTA-TGA-GAA-TCT-CG	*cqsS* (*b1775*)
***cqsS*R**	ATC-AGA-AAG-GCA-CCG-CAG-G	
***hcp1*F**	GGG-CAT-GGT-GGC-TTT-AAG-C	(*b0573*)
***hcp1*R**	CGC-ACC-ATC-CAC-TTG-TTT-TG	
***luxS*F**	CCT-GAT-CGG-AAC-CCC-TTC-A	*luxS* (*a0117*)
***luxS*R**	GCC-GCT-AAC-CAA-GAA-TCA-GC	
***ompU*F**	GCG-ACA-CCC-TAG-AGC-ATC-GT	*ompU* (*a1598*)
***ompU*R**	CGA-AGT-TAC-CGC-CGA-TAC-CA	
***pilP*F**	TGC-GAA-GTA-AGC-AGA-AAG-GGA	putative Pilus assembly protein PilP (*a2966)*
***pilP*R**	CCA-ACA-GCC-ACA-CTC-CAA-CC	
***virB4*F**	GTG-AGC-TGG-AGC-GGA-TAA-GC	putative Type IV secretion system protein B4 (*a3554*)
***virB4*R**	GTA-TGA-CAG-CCA-CTC-CCC-GT	
***sypA*F**	AAC-ATA-ACC-GCA-GCA-TGC-G	Anti-anti-sigma regulatory factor (*b1520*)
***sypA*R**	TTT-CAA-GTG-GCT-GCC-CCT-T	
